# Gender Differences in the Associations of Plasma Pyridoxal 5′-Phosphate with Plasma Polyunsaturated Fatty Acids among US Young and Middle-Aged Adults: NHANES 2003–2004

**DOI:** 10.3390/nu13020477

**Published:** 2021-01-31

**Authors:** Hyojung Kim, Evelyn B. Enrione, Vijaya Narayanan, Tan Li, Adriana Campa

**Affiliations:** 1 Department of Dietetics and Nutrition, Robert Stempel College of Public Health and Social Work, Florida International University, Miami, FL 33199, USA; hkim030@fiu.edu (H.K.); enrionee@fiu.edu (E.B.E.); vijaya.narayanan@fiu.edu (V.N.); 2 Department of Biostatistics, Robert Stempel College of Public Health and Social Work, Florida International University, Miami, FL 33199, USA; tanli@fiu.edu

**Keywords:** gender, vitamin B6, pyridoxal 5′-phosphate, polyunsaturated fatty acid, NHANES

## Abstract

Vitamin B6-restricted diets and low plasma pyridoxal 5′-phosphate (PLP) status altered plasma polyunsaturated fatty acids (PUFA) compositions. Evidence suggests the role of gender in the metabolism of vitamin B6 and PUFA. However, no epidemiologic study examined the impact of gender on the relationship between vitamin B6 and PUFA status in adults. Thus, we investigated whether there were gender differences in the association of vitamin B6 intake and plasma PLP concentration with plasma PUFA concentrations and ratios (eicosapentaenoic acid (EPA), docosahexaenoic acid (DHA), arachidonic acid (AA), EPA + DHA, EPA/AA, (EPA + DHA)/AA) in US young/middle-aged adults. In total, 864 participants (20–59 years; 484 men, 380 women) from the National Health and Nutrition Examination Survey (NHANES) 2003–2004 were used for this cross-sectional study. Nutrient intakes were estimated from two 24 h recalls and supplement questionnaires; plasma PLP and PUFA were measured. Multivariate linear regression was utilized to obtain unstandardized (b) and standardized (β) coefficients. Covariates included demographic, socioeconomic, dietary variables, physical activity level, cigarette smoking status, alcohol consumption, prescription medication use, and BMI. There were significant interactions between gender and PLP on EPA (*P-_interaction_* = 0.004), DHA (*P-_interaction_* = 0.020), EPA + DHA (*P-_interaction_* = 0.010), EPA/AA (*P-_interaction_* = 0.002), (EPA + DHA)/AA (*P-_interaction_* = 0.004), whereas no interaction between gender and B6 intake existed. In gender-stratified analyses, in men, PLP was positively associated with EPA (β = 0.138, b = 0.104, *p* = 0.0004), DHA (β = 0.101, b = 0.058, *p* = 0.036), EPA + DHA (β = 0.125, b = 0.073, *p* = 0.005), EPA/AA (β = 0.144, b = 0.099, *p* = 0.0002), (EPA + DHA)/AA (β = 0.123, b = 0.068, *p* = 0.005). However, no associations between PLP and PUFA existed in women. In conclusion, gender differences were found in the relationships between plasma PLP and plasma EPA, DHA, EPA + DHA, EPA/AA, and (EPA + DHA)/AA, with significant direct associations in men only among US young/middle-aged adults.

## 1. Introduction 

The interrelationship between vitamin B6 (B6) and unsaturated fatty acid metabolism was recognized in the 1930s [1,2,3]. Since then, animal studies have demonstrated that vitamin B6 deficiency alters plasma and tissue n-3 and n-6 polyunsaturated fatty acids (PUFA) profiles, suggesting the potential metabolic link between vitamin B6 and PUFA [4,5,6,7,8]. In male rats fed B6-restricted diets, linoleic acid (LA; 18:2n-6) increased, but arachidonic acid (AA; 20:4n-6) decreased in plasma and liver phospholipids compared to controls [4]. Similarly, another study reported an increase in LA and a decrease in AA in liver microsomal and plasma total lipids in B6-deficient male rats [7]. Tsuge et al. [8] further showed the lower contents of eicosapentaenoic acid (EPA; 20:5n-3), docosahexaenoic acid (DHA; 22:6n-3), and AA in plasma total lipids in B6-deficient male rats compared to pair-fed controls. Moreover, epidemiologic studies have indicated that low levels of circulating pyridoxal 5′-phosphate (PLP) change the compositions of PUFA in serum or plasma [9,10,11,12]. A clinical study with 23 healthy men and women combined (20–40 years) found that marginal vitamin B6 deficiency (plasma PLP concentration between 20 and 30 nmol/L), which was induced by a 28 day B6-restricted diet, reduced plasma concentrations of EPA, DHA, and AA and increased slightly plasma n-6/n-3 PUFA ratio [10]. Thus, the data from animal and human studies thus far suggest that vitamin B6 deficiency may adversely affect PUFA compositions [4,5,6,7,8,9,10,11,12].

PUFA, such as EPA, DHA, and AA, provide beneficial functions as important constituents of cell membrane for human development and optimal health [13,14,15,16] and play a critical role in immunomodulatory function in humans [13,15,16,17]. Gender may influence the endogenous synthesis of n-3 and n-6 PUFA [18,19,20,21,22,23], and women may have a higher metabolic capacity to convert α-linolenic acid (ALA; 18:3n-3) into longer chain n-3 PUFA in blood lipids than men [13,22,23,24]. Two stable isotope studies [22,23] on fatty acid metabolism with six young women (28 ± 4 years) [22] and with six young men (27–40 years) [23] reported that, in women, the estimated net fractional interconversion of the tracer [U-^13^C]ALA to EPA was 21%, and to DHA was 9% [22]. In contrast, in men, the estimated values were 8% for EPA and non-detectable for DHA, implying the inhibition or the restriction of DHA synthesis for men [23]. Similarly, a cross-sectional study of the 1997 National Nutrition Survey with 2793 New Zealanders (≥15 years) showed a higher proportion of DHA but a lower EPA in serum phospholipids in women than in men [18]. Further, a recent meta-analysis found higher contributions of DHA and AA to plasma total lipids and plasma phospholipids in women than in men [14].

Based on the described evidence regarding the interaction between vitamin B6 and PUFA metabolism and the differential PUFA compositions by gender, it may be plausible that gender may contribute to the relationship between PUFA levels, vitamin B6 intake, and B6 status, measured by PLP. Both vitamin B6 status [25,26] and PUFA levels such as EPA and DHA [15,27] have been shown to be inversely associated with inflammation; it has been suggested that gender differences exist in inflammatory diseases such as cardiovascular diseases [28,29,30]. Thus, understanding the relationship between gender, vitamin B6, and PUFA has important public health implications. However, data indicating the interconnection between gender, vitamin B6, and PUFA are sparse. Therefore, we aimed to investigate whether the association of vitamin B6 intake and plasma PLP level, respectively, with plasma PUFA levels differed by gender in a large representative sample of adults aged 20–59 years from the National Health and Nutrition Examination Survey (NHANES) 2003–2004. 

## 2. Materials and Methods 

### 2.1. Data Source and Study Population

This study is a cross-sectional study utilizing the NHANES data, which follow a stratified, multistage, clustered probability sampling design. The 2003–2004 cycle was utilized since all the main independent and dependent variables of interest were available only in this dataset (especially plasma PLP and plasma PUFA). The NHANES, conducted by the National Center for Health Statistics (NCHS), is designed to obtain nationally representative information on the health and nutritional status of the civilian non-institutionalized US population [31]. 

Written informed consent was obtained from all participants [32], and the survey protocol was approved by the Research Ethics Review Board of the NCHS [33]. Participants were interviewed by trained staff for collecting household interview data, including demographic, socioeconomic, dietary supplements, and prescription drug data, via a computer-assisted personal interview (CAPI) system [32]. The participants were invited to the mobile examination center (MEC), where anthropometric, reproductive history, and other health-related data were collected [32]. In the MEC laboratory, blood specimens were processed and shipped to corresponding laboratories for analysis [34]. 

From the total 10,122 participants in the NHANES 2003–2004, participants aged ≥20 years with fasting (≥8 h) blood samples were used in this study (*n =* 4034). Of these, 1829 participants had measurements of plasma concentrations of ALA, LA, EPA, DHA, or AA. Of these, participants aged 20–59 years numbered 1141. The data from adults aged 20–59 years were used since the aging process may alter lipid metabolism by reducing fat oxidation and free fatty acid mobilization [35,36] and changing blood PUFA levels [37,38,39].

We excluded participants with dietary recalls which were unreliable or not met the minimum criteria (*n =* 3). Plasma PLP level may be influenced by diabetes [40], liver diseases [40], the current uses of oral contraceptives (OC) [40,41], and hormone replacement therapy (HRT) [41]; plasma PUFA levels may also be altered by the uses of oral [21] or injectable [42] (e.g., Depo-Provera) contraceptives and HRT [43]. For this reason, participants with physician-diagnosed diabetes or hemoglobin A1C (A1C) ≥6.5% or fasting plasma glucose ≥126 mg/dL (*n =* 76) or current liver diseases (*n =* 20), current use of HRT (*n =* 39), or oral/injectable contraceptives (*n =* 52) were excluded. Pregnant (*n =* 87) or lactating (*n =* 15) women were also excluded since hormonal changes may lower plasma PLP levels during pregnancy and lactation [40] and may alter plasma PUFA levels during pregnancy [44,45].

**Figure 1:**
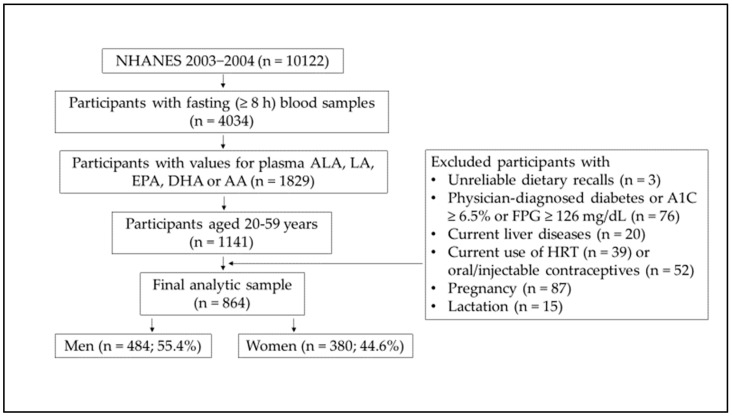
Flow chart of National Health and Nutrition Examination Survey (NHANES) 2003–2004 analytic sample. A1C, hemoglobin A1C; FPG, fasting plasma glucose; HRT, hormone replacement therapy; %, sample-weighted percentage; sample sizes (*n*) were presented as unweighted.

Consequently, the final resulting analytic sample size was 864 participants (484 men, 380 women). Depending on nonpositive weights, missing data (the responses of “refused” or “don’t know” were treated as missing), and/or model covariates, the samples for descriptive and regression analyses were available from 674 to 864 participants. Of the final analytic sample, the proportion of men was higher than women (55.4% for men vs. 44.6% for women), which came from excluding women with the current use of HRT, oral/injectable contraceptives, or with pregnancy or lactation. A flow chart describing the sample selection is presented in Figure 1. 

### 2.2. Assessment of Vitamin B6 and PUFA Intakes from Food and Supplements

According to the NHANES documentation [46], collection of two days of dietary intake data was performed by trained staff; a first 24 h recall interview at MEC was used for the first day’s data and a second 24 h recall interview by telephone 3–10 d after the MEC examination for the second day’s data. Participants completed both dietary interviews using the US Department of Agriculture’s Automated Multiple-Pass Method (AMPM) [46]. 

Personal interview data on the use of vitamins, minerals, and other dietary supplements (supplement name, ingredients, amounts, and serving size) were recorded by the interviewer [47]. Participants were asked duration, frequency, and daily amount in the past 30 days of dietary supplement use [48]. NCHS obtained product label information for dietary supplements from the manufacturer/retailer, the Internet, company catalogs, and the Dietary Supplement Label Database (DSLD) containing the full label contents from supplement products in the US [48]. The ingredient amounts in supplement products were determined by matching the name and the manufacturer of the product to those in a database developed by NCHS and the National Institutes of Health (NIH)’s Office of Dietary Supplements [48]. Using various dietary supplement files, we identified participants who reported the use of any dietary supplements containing the ingredients of vitamin B6, EPA, DHA, and ALA. The average daily intake of these nutrients from dietary supplements was estimated for participants, using the number of days that the use of supplements was reported, the amount taken per day, and the serving size unit from the supplement product label. 

The mean dietary daily intakes of vitamin B6, ALA, LA, EPA, DHA, AA, total fat, and total energy were calculated from the averages of both the first and the second day dietary recall interviews. Octadecatrienoic acid (linolenic acid; 18:3), which includes mainly α-linolenic acid and lesser quantities of γ-linolenic acid (18:3n-6) [49], was examined. Octadecadienoic acid (LA), eicosatetraenoic acid (AA), EPA, and DHA were also assessed. Total daily intakes of vitamin B6, EPA, DHA, and ALA from food and dietary supplements were estimated by combining the mean dietary daily intake from dietary recall data and the average daily intake from dietary supplement data. 

### 2.3. Definition of Gender 

The terms of gender and sex are often used interchangeably in the scientific literature [50,51]. Sex is related to the biological distinctions that differ between men and women based on reproductive functions. In contrast, gender refers to socially and culturally constructed differences between men and women on the basis of the sex of the individual or the personal identification of an individual’s own gender [50,51]. Based on the usage of gender in the codebook of NHNAES [52], the term of gender was used in this study, although this study’s possible suggested mechanisms relevant to the function of vitamin B6 and PUFA are inherent to the biological differences between men and women. 

### 2.4. Laboratory Measurements of Plasma PLP and PUFA

#### 2.4.1. Pyridoxal 5′-Phosphate (PLP) 

Homogeneous, nonradioactive, enzymatic assay (A/C Diagnostics, San Diego, CA, USA) was used for measuring plasma PLP concentration (nmol/L) [53]. The mean intra-assay coefficient of variation (CV) from 7.8% to 8.3% and the mean inter-assay CV from 12.0% to 13.1% were reported [53]. Assay values below the lower limit of detection were replaced with 7.1 nmol/L (the detection limit of 10.09 nmol/L divided by the square root of 2) [53,54]. Plasma PLP was dichotomized by 20 nmol/L as a categorical variable because plasma PLP ≥ 20 nmol/L is the definition of vitamin B6 adequacy used to set the current estimated average requirements (EAR) and recommended dietary allowances (RDA) of vitamin B6 [55]. 

#### 2.4.2. Plasma Polyunsaturated Fatty Acids (PUFA)

Blood samples for fatty acid concentration measurements (µmol/L) were collected from participants ≥20 years after fasting for ≥8 h. Gas chromatography-mass spectrometry was used to measure plasma fatty acids. Modified Lagerstedt et al. method was performed to measure plasma total fatty acid concentrations [56]. The mean intra-assay CV (standard deviation (SD)) for analytes was reported as 9% (10%) and the mean inter-assay CV (SD) for analytes as 8% (10%) [56]. In this study, we excluded subjects missing data on any individual fatty acids since this would influence the calculation of the sum of EPA and DHA and the ratios of EPA/AA and (EPA + DHA)/AA [57]. 

### 2.5. Study Covariates 

Since plasma PUFA levels may be influenced by the following demographic, socioeconomic variables, physical activity level, cigarette smoking status, alcohol consumption, prescription medication use, BMI [58,59,60], and menopausal status [61], they were selected as covariates in this study. In addition, since the consumption of fatty acids and total fat can influence PUFA profiles [57,62,63], dietary factors including total fat intake and PUFA intake were included as model covariates.

#### 2.5.1. Demographic Factors

Age was categorized as 20–29, 30–39, 40–49, and 50–59 years. Race/ethnicity was categorized into non-Hispanic White, non-Hispanic Black, Hispanic (Mexican American, Other Hispanic), and Others. 

#### 2.5.2. Socioeconomic Factors

Family poverty income ratio (PIR), an index for the ratio of annual family income to the poverty threshold, was dichotomized by 1.3. Participants with PIR below 1.3 are eligible for the Supplemental Nutrition Assistance Program [64]. Educational attainment was categorized into high school graduation or less and some college or higher. 

#### 2.5.3. Dietary Factors

Dietary variables, such as total intakes of vitamin B6, EPA, DHA, ALA from food and supplements, dietary intakes of LA and AA from food, and total fat intake from food, were used as covariates. 

#### 2.5.4. Other Factors 

Cigarette smoking status was categorized as a never smoker who smoked <100 cigarettes in life; a former smoker who did not smoke at the time of interview among those who had smoked ≥100 cigarettes in life; and a current smoker who reported ongoing smoking. Alcohol consumption was categorized as an abstainer who had <12 drinks of any type of alcoholic beverage in life; a former drinker who had ≥12 drinks in life or any one year, but none in the past 12 months; a current drinker who had ≥12 drinks in life, and drank ≥1 time in the past 12 months. 

Physical activity level expressed on the metabolic equivalent of task (MET) score was categorized as <500, 500–1000, and ≥1000 MET min/week, which was calculated from the frequency and the duration of household/yard work, transportation, and leisure time. Prescription medication use was defined as a positive response to the question of taking any prescription medication in the past month. 

Body mass index (BMI) was categorized as <18.5, 18.5–24.9, 25–29.9, and ≥30 kg/m^2^. Menopause was defined to be when a woman did not have a menstrual period in the past 12 months.

### 2.6. Statistical Methods 

Appropriate sample weights were applied to account for complex survey design and the unequal probability of selection, noncoverage, and nonresponse bias. Variance estimates were computed using Taylor series linearization accounting for the complex sample design. All tests were two-sided, and the significance level was set at *p* < 0.05. Statistical analyses were performed with SAS 9.4 (SAS Institute Inc., Cary, NC, USA). 

The dietary and the total intakes of vitamin B6, ALA, LA, EPA, DHA, and AA were energy-adjusted using the residual method to employ the regression model with total caloric intake as the independent variable and absolute nutrient intake as the dependent variable [65]. 

Plasma PLP and PUFA variables were natural log-transformed to improve normality due to skewed distributions as they were used as dependent variables. We tested the normality of the distributions of plasma PLP and PUFA (EPA, DHA, AA, EPA + DHA, EPA/AA, (EPA + DHA)/AA) variables using quantile-quantile (Q-Q) plots and skewness values in the univariate analysis. Those variables were highly skewed except plasma AA, which were slightly skewed. To improve normality, plasma PLP and PUFA variables were natural log-transformed. After the transformation, we tested the normality using the same method described above, and those variables improved normality, approaching near-normal. The use of log-transformed AA did not make significant differences in the findings compared with the use of the original metric AA.

For descriptive statistics of the aforementioned covariates for all study participants, men and women each, we estimated frequencies and sample-weighted percentages with standard errors (SE) for categorical variables and arithmetic means (nutrient intake variables) and geometric means (plasma variables) with SE for continuous variables. The characteristics between men and women were compared using Rao-Scott F-adjusted chi-square tests for categorical variables and *t*-tests for continuous variables. 

Plasma PLP status is determined by vitamin B6 intake [12,40,41]; there was a positive correlation between total vitamin B6 intake and plasma PLP in all participants (correlation coefficient (ρ) = 0.26), men (ρ = 0.24) and women (ρ = 0.30), respectively, in this study (Table S1). 

If vitamin B6 intake and plasma PLP are included together as independent variables in the model, this may lead to the unreliable estimation of regression coefficients in the fitted model due to possible multicollinearity [66]. In addition, the inclusion of B6 intake and PLP together as independent variables did not make material differences in the findings. Therefore, reported findings were from regressions using two separate models for vitamin B6 intake and plasma PLP, respectively. 

To test whether gender modifies the relationship between vitamin B6 intake and plasma PLP, respectively, and plasma PUFA, we added the interaction terms of gender*B6 intake and gender*PLP each in the fully adjusted model before performing the gender-stratified analysis. While the gender*B6 intake interaction was not significant, the gender*PLP interaction was significant. Thus, we evaluated the relationship between PLP and plasma PUFA using gender-stratified analysis. 

Gender-stratified bivariate and multivariate linear regression analyses were performed to examine whether the association of plasma PLP concentration with plasma PUFA concentrations (EPA, DHA, AA, EPA + DHA) and ratios (EPA/AA, (EPA + DHA)/AA) differed by gender. Natural log-transformed plasma PLP and PUFA variables of interest were used in regression models after inspecting residual plots of gender-stratified models. 

In the gender-specific models, the covariates were sequentially introduced in the following models. Model 0 was unadjusted. Model 1 was adjusted for demographic variables (age, race/ethnicity), BMI, dietary variables (total intakes of vitamin B6, EPA, DHA, ALA from food and supplements, dietary intake of LA and AA from food, total fat intake from food), and menopausal status (only for women). Model 2 was adjusted for all variables in model 2, plus socioeconomic variables (PIR, educational attainment), physical activity level, cigarette smoking status, alcohol consumption, and prescription medication use.

Unstandardized regression coefficients (b) with 95% confidence intervals were estimated. Standardized regression coefficient (β) was employed to express the change in log-transformed plasma PUFA concentrations and ratios in SD for 1 SD of change in log-transformed plasma PLP concentration. For each model, the coefficient of determination, R^2^, was quantified to measure the percentage of the total variability in plasma PUFA concentrations and ratios that the model explains. In the adjusted models 1 and 2, R^2^ was adjusted for the model’s number of predictors.

No multicollinearities were detected among independent variables in the fully adjusted gender-stratified regression models (for men: variance inflation factors (VIF) = 6.9 for total EPA intake, VIF = 7.3 for total DHA intake, VIF < 2.3 for the other variables; for women: VIF = 6.2 for total EPA intake, VIF = 7.4 for total DHA intake, VIF < 3.5 for the other variables).

## 3. Results

### 3.1. Demographic, Socioeconomic, and Other Characteristics of Participants by Gender 

Table 1 presents the participants’ characteristics by gender. There were more men than women in the study population (*p* = 0.003). Over one-third of women were in the range of 40–49 years of age, with more than a quarter of men in the range of 40–49 years of age (*p* = 0.046). Gender was distributed similarly for non-Hispanic White, and there was a tendency of a higher proportion of men for Hispanic and Other groups and a lower proportion of men for non-Hispanic Black compared to women (*p* = 0.09). The prevalence of overweight was higher for men than for women, while women were more likely to be obese than men (*p* = 0.0004). Compared with men, women were more likely to have a low family income (*p =* 0.0496) and were less likely to consume alcohol (*p* = 0.002). Men tended to be more physically active than women (*p* = 0.06). More women used vitamin B6 supplements (*p* = 0.018) and prescription medications (*p* = 0.011) than men. Approximately one-fifth of the women were postmenopausal (*p* < 0.0001). On the other hand, there were no differences between men and women for educational attainment, cigarette smoking status, and n-3 PUFA supplement use. 

### 3.2. Distributions of the Intakes of Vitamin B6 and PUFA and the Concentrations of Plasma PUFA and PLP by Gender

Table 2 displays distributions of the energy-adjusted dietary (from food) and total intakes (from food and supplements) of vitamin B6 and PUFA, concentrations and ratios of plasma PUFA, and concentrations of PLP by gender. Distributions of the original metric nutrient intakes were presented in Table S2. Using the residual method [65], nutrient intakes were evaluated in relation to the mean total energy intake of all study participants; the resulting measures of energy-adjusted nutrient intakes were independent of total energy intake. Due to the application of this method, overall, the energy-adjustment resulted in changes in the mean dietary intake estimates of the nutrients, with the decreased energy-adjusted mean values from the original metric mean values in men and the increased energy-adjusted means from the original metric means in women. The original metric dietary vitamin B6 intake was lower in women than in men, but the energy-adjusted dietary vitamin B6 intake was similar between them, although the difference was not significant. A similar pattern was observed in the case of total vitamin B6 intake.

There was a large increase from 2 mg of dietary vitamin B6 to 5 mg of total vitamin B6 from food and supplements, indicating some participants were taking high dosages of vitamin B6 supplements. We observed that, among vitamin B6 supplement users (*n =* 222 without 21 nonpositive dietary sample weight), the participants greater than 95 percentiles consumed more than 58.3 mg/d of total vitamin B6 up to 187.0 mg/d (median: 2.5 mg/d, interquartile range (IQR): 1.5–7.6 mg/d; data not shown), which could affect the large increase. Meanwhile, men and women had similar total vitamin B6 intake values from food and supplements, although a higher proportion of women took vitamin B6 supplements than men. This can be explained by the greater proportion of high dosage of vitamin B6 supplement intake in men than in women. For example, among vitamin B6 supplement users, six men and two women consumed more than 100 mg/d of vitamin B6 supplements, with five extreme observations of four men and one woman (149.4, 149.5, 150.7, 152.6, 187.0 mg/d; data not shown).

A mean total energy intake was lower in women than in men (*p* < 0.0001) after adjusting for demographic factors (age, race/ethnicity), socioeconomic factors (PIR, educational attainment), physical activity level, cigarette smoking status, alcohol consumption, prescription medication use, BMI, and menopausal status. A mean dietary ALA intake was higher in women than in men (*p* = 0.046), whereas mean dietary EPA, DHA, and AA intakes were lower in women than men (*p* = 0.022, *p* = 0.027, *p* = 0.019, respectively). Similarly, a mean total ALA intake from food and supplements was higher in women (*p* = 0.036), but a mean total DHA intake tended to be greater in men than in women (*p* = 0.06). Differences between men and women did not exist in dietary intakes of vitamin B6 and LA from food and total intakes of vitamin B6 and EPA from food and supplements.

Higher geometric means of plasma DHA concentration and (EPA + DHA)/AA ratio were observed in women than in men (*p* = 0.043, *p* = 0.034 each) after adjusting for demographic and socioeconomic factors, total fat intake, total intakes of EPA, DHA, ALA, dietary intakes of LA and AA, BMI, physical activity level, cigarette smoking status, alcohol consumption, prescription medication use, and menopausal status. A geometric mean plasma EPA concentration tended to be higher in men than in women (*p* = 0.09). There were no differences between men and women in plasma concentrations of ALA, LA, AA, EPA + DHA, and EPA/AA ratio.

A geometric mean plasma PLP concentration was lower in women than in men after adjusting for demographic and socioeconomic factors, total energy intake, total vitamin B6 intake, BMI, physical activity level, cigarette smoking status, alcohol consumption, prescription medication use, and menopausal status (*p* = 0.0002). Vitamin B6 deficiency, defined by plasma PLP concentration <20 nmol/L, was more common among women than men, with more than a quarter of women with vitamin B6 deficiency (*p* < 0.0001).

### 3.3. No Interaction Effects between Gender and Vitamin B6 Intake on Plasma PUFA Concentrations and Ratios 

To assess whether gender modifies the association between vitamin B6 intake and plasma PUFA, we included an interaction term of gender*B6 intake in multivariate linear regression models fully adjusted for demographic and socioeconomic factors, total fat intake, total intakes of EPA, DHA, and ALA, dietary intakes of LA and AA, BMI, physical activity level, cigarette smoking status, alcohol consumption, prescription medication use, and menopausal status. There was no significant interaction between gender and vitamin B6 intake on EPA (*P-_interaction_* = 0.37), DHA (*P-_interaction_* = 0.11), AA (*P-_interaction_* = 0.86), EPA + DHA (*P-_interaction_* = 0.14), EPA/AA (*P-_interaction_* = 0.34), and (EPA + DHA)/AA (*P-_interaction_* = 0.11), respectively (data not shown). These results indicate that the relationship between vitamin B6 intake and plasma EPA, DHA, AA, EPA + DHA, EPA/AA, (EPA + DHA)/AA did not differ between men and women.

### 3.4. Associations of Plasma PLP Concentration with Plasma PUFA Concentrations and Ratios, Stratified by Gender

There was a significant interaction between gender and PLP on EPA (*P-_interaction_* = 0.004), DHA (*P-_interaction_* = 0.020), EPA + DHA (*P-_interaction_* = 0.010), EPA/AA (*P-_interaction_* = 0.002), and (EPA + DHA)/AA (*P-_interaction_* = 0.004), respectively, but not AA (*P-_interaction_* = 0.37), in the fully adjusted model, indicating that the association between PLP and EPA, DHA, EPA + DHA, EPA/AA, and (EPA + DHA)/AA, but not AA, differs by gender (Table 3). 

The relationships between plasma PLP concentration and plasma PUFA concentrations and ratios by gender are presented in Table 3. In gender-stratified bivariate and multivariate regression models, plasma PLP was positively associated with plasma EPA, DHA, EPA + DHA, EPA/AA, (EPA + DHA)/AA, respectively, in men only. In the fully adjusted model 2, the association of PLP with EPA (b = 0.104, *p* = 0.0004), DHA (b = 0.058, *p* = 0.036), EPA + DHA (b = 0.073, *p* = 0.005), EPA/AA (b = 0.099, *p* = 0.0002), and (EPA + DHA)/AA (b = 0.068, *p* = 0.005) were significant. In contrast, there were no significant associations between plasma PLP and EPA, DHA, EPA + DHA, EPA/AA, and (EPA + DHA)/AA in women.

It is interpreted that, among men, the log plasma concentrations of EPA, DHA, EPA + DHA and ratios of EPA/AA and (EPA + DHA)/AA increase by 0.138 SD, 0.101 SD, 0.125 SD, 0.144 SD, and 0.123 SD, respectively, for 1 SD increase in log plasma PLP concentration in the full model 2. Based on the regression coefficients for men, the estimated effects of PLP on PUFA changed between the unadjusted model 0 and the full model 2. In men, the standardized coefficient for EPA fell from 0.203 in model 0 to 0.138 in model 2, for DHA from 0.169 to 0.101, for EPA + DHA from 0.198 to 0.125, for EPA/AA from 0.215 to 0.144, and for (EPA + DHA)/AA from 0.198 to 0.123. These suggest that demographic, socioeconomic, dietary (total fat intake, total intakes of EPA, DHA, and ALA, dietary intakes of LA and AA) factors, physical activity level, cigarette smoking status, alcohol consumption, prescription medication use, and BMI may mediate the association between PLP and EPA, DHA, EPA, EPA + DHA, EPA/AA, and (EPA + DHA)/AA, respectively, among men.

Based on the results of the coefficient of determination, adjusted R^2^ (aR^2^), for men in the partial model 1, 24%, 27%, 29%, 29%, and 32% of the variance in EPA, DHA, EPA + DHA, EPA/AA, and (EPA + DHA)/AA, respectively, is explained by demographic, dietary factors and BMI. After further adjustment in the full model 2, in men, the additional 3%, 4%, 3%, 3%, 4% of the variance in EPA, DHA, EPA + DHA, EPA/AA, (EPA + DHA)/AA each is further explained by socioeconomic factors, physical activity level, cigarette smoking status, alcohol consumption, and prescription medication use, in addition to the model 1 covariates (aR^2^ = 0.27, aR^2^ = 0.31, aR^2^ = 0.32. aR^2^ = 0.32, aR^2^ = 0.36, respectively). 

Lastly, the relationship between PLP and AA was not affected by gender. No significant association existed between PLP and AA in all models for both men and women. 

## 4. Discussion 

This present study observed gender differences in the relationships between plasma PLP and plasma EPA, DHA, EPA + DHA, EPA/AA, and (EPA + DHA)/AA, with the significant positive associations in men only, but not in women, among US young and middle-aged adults after adjusting for demographic, socioeconomic and dietary factors, physical activity level, cigarette smoking status, alcohol consumption, prescription medication use, and BMI. In addition, the association between vitamin B6 intake and the selected PUFA was not affected by gender. To the best of our knowledge, this study is the first population-level observational study to report the gender differences in the relationship between vitamin B6 status, measured by plasma PLP, and plasma PUFA levels in adults aged 20–59 years.

Compared with men, a mean plasma DHA concentration was higher in women, which is supported by the studies describing that women had higher contributions of DHA to plasma lipids than men [14,20,24]. In addition, vitamin B6 intake was similar in both men and women in this study, but the mean plasma PLP level was lower in women than in men (33.5 nmol/L for women, 51.1 nmol/L for men). The differences in plasma PLP levels between them may be partially attributable to the prevalence of vitamin B6 deficiency (defined by plasma PLP < 20 nmol/L) for women, which was approximately three times higher than for men (29.0% for women, 10.4% for men).

Vitamin B6 serves as a coenzyme in the form of PLP for various metabolic reactions, including the synthesis of hemoglobin and neurotransmitters, interconversion of amino acids, gluconeogenesis, and metabolism of tryptophan, one-carbon units, and nucleic acids [67,68,69]. PUFA, such as EPA, DHA, and AA, provide important structural components of cell membranes [14,15] and exert immunomodulatory functions in humans [15,17]. EPA (n-3 PUFA) and AA (n-6 PUFA) can be synthesized from the precursors, ALA and LA, respectively, in the series of desaturation and elongation reactions catalyzed by elongases and desaturases such as Δ6-desaturase (D6D) and Δ5 desaturase (D5D) [16,24]. DHA is further converted from docosapentaenoic acid (DPA; 22:5n-3) via chain elongation, desaturation (by D6D), and peroxisomal β-oxidation [24]. D6D is the rate-limiting step for the biosynthesis of longer chain PUFA, including EPA and DHA; it is influenced by PLP status [12,70,71]. Although the underlying mechanisms of how vitamin B6 regulates D6D has not been fully understood yet [71], it may be speculated that inadequate vitamin B6 status could result in lowering the activity of D6D, thereby leading to the altered compositions of n-3 and n-6 PUFA. 

An animal study showed that the D6D activity in male rats fed B6-deficient diets was lower than in the pair-fed control, suggesting low vitamin B6 status disturbed metabolic conversions from LA to AA and from ALA to EPA and DHA, with more reduction in DHA [8]. An in vitro study demonstrated that the relative mRNA expressions of FADS2 (D6D) and FADS1 (D5D) genes were lower in vitamin B6-restricted human hepatoma cells than in control [71]. Further, a case-control study with healthy men and women (21–60 years) reported that D6D activity plus D5D activity (EPA/ALA; enzyme activity measured by a product/precursor ratio) and the second D6D activity (DHA/DPA) were lower in a group with low serum vitamin B6 status (pyridoxine + PLP <3 μg/L; *n =* 21) than in a control group with normal B6 status (*n =* 22), although plasma PUFA compositions in the group with low B6 status were not different from the control group [9]. These previous findings imply that vitamin B6 status may influence fatty acid desaturation, possibly via PLP-dependent D6D catalyzing PUFA synthesis [5,8,9,71]. Therefore, in part, these may explain the positive associations between plasma PLP and PUFA for men in this study (Table 3). 

This study revealed the significant positive associations between plasma PLP and plasma EPA, DHA, EPA/AA, and (EPA + DHA)/AA in men alone, not in women. D6D activity may be regulated by nutritional and non-nutritional factors, including glucose, alcohol, age, estrogen, insulin, etc. [24,72,73]. Besides, the status of iron, which is located at every terminal protein of the D6D enzyme complex [74], was shown to influence the activity of D6D [75,76,77,78]. Evidence from animal and human studies has indicated that low iron levels, such as iron depletion or iron deficiency, may adversely affect PUFA synthesis [75,76,77,78,79]. It is noteworthy that the aforementioned study by Krajcovicova-Kudlackova et al. [9] demonstrated that the D6D activity (EPA/ALA, DHA/DPA) was positively correlated with serum iron levels. Moreover, the loss of D6D activity and the inhibition effect on PUFA synthesis were more pronounced in the group with low serum iron levels (<12 μmol/L for men, <10 μmol/L for women; *n =* 16) than in the group with low vitamin B6 level. Although data on the relationship between iron and PUFA metabolism are limited, the evidence thus far suggests that low iron status as well as low vitamin B6 status might negatively affect PUFA metabolism [9,75,76,77,78,79,80]. Furthermore, the present study showed that, compared with men, women had not only the lower mean plasma PLP concentration but also the low serum iron concentration, with the greater prevalence of low vitamin B6 and iron status for women (Table 2; Table S3), which is in agreement with the studies showing the greater prevalence of low iron status in women than in men [81,82]. Thus, the non-significant association of vitamin B6 status and PUFA in women, unlike men, in this study might be explained possibly due to the interaction between iron and PUFA metabolism [75,76,77,78,79]. The combination of vitamin B6 deficiency and low iron status might impact much more adversely on PUFA synthesis in women than in men. To confirm this, future research is necessary to explore whether iron could be another contributing factor to the gender differences in the relationship between vitamin B6 and PUFA metabolism. 

Since plasma PLP can be reduced by inflammation [40], the differences in plasma PLP concentration between men and women may be, in part, mediated by inflammation. The proportion of elevated C-reactive protein (CRP) concentration (≥3 mg/L CRP), indicative of inflammation [83], was higher in women than in men (data not shown).

In particular, EPA and AA may play a critical role in regulating inflammatory responses by serving as precursors of EPA-derived and AA-derived eicosanoids, respectively [15]. AA promotes platelet aggregation and inflammatory reactions, whereas EPA exerts anti-inflammatory effects and acts as an antagonistic regulator against AA [15]. EPA and DHA also generate resolvins, which have anti-inflammatory and inflammation-resolving properties [15]. Further, circulating EPA level was inversely associated with inflammatory markers and cardiovascular disease (CVD) incidence [27]; plasma EPA/AA is considered predictive of CVD risk [84,85] and chronic inflammation, with a higher EPA/AA ratio corresponding to lower levels of inflammation [85]. Besides, vitamin B6 status was inversely associated with inflammation [25,26]. This study revealed significant positive associations between plasma PLP and EPA, DHA, and EPA/AA in men (Table 3), which may imply a possible interrelationship between vitamin B6 status and blood PUFA levels and inflammatory diseases in men. However, the interconnection among vitamin B6, PUFA, and inflammation is unclear, and it remains to be answered whether inflammation in relation to vitamin B6 and PUFA would differ by gender.

This study has several limitations. First, there are remaining suspected confounders that were not measured to examine the association between plasma PLP and plasma PUFA. This study did not include lifestyle habits, such as vegetarianism, veganism, folate, vitamin B12, homocysteine, and methylmalonic acid in the assessment. Vegetarianism and veganism may influence plasma PUFA concentrations, and the adherence to such diets may increase the risk of developing vitamin B12 deficiency [86] and lower n-3 PUFA [87]. Vitamin B6 participates in one-carbon metabolism, and the associated metabolites, such as folate, vitamin B12, and homocysteine, are relevant to vitamin B6 deficiency [40]. For this reason, the observed association could still be influenced by residual confounding effects. 

Second, in NHANES, fatty acids in plasma and serum were measured in two survey cycles, 2003–2004 and 2011–2012, respectively, while plasma or serum PLP was measured in four different cycles, 2003–2004, 2005–2006, 2007–2008, and 2009–2010. Thus, we utilized the data of fatty acids and PLP from the 2003–2004 cycle, which collected both of them together. However, since, over the past two decades, the use of fish oil supplements increased in US adults [88], it is possible that this could impact the population’s level of PUFA status. Besides, in the 2003–2004 cycle, plasma fatty acids were measured as part of a surplus specimen project. Only about 45% of the participants with fasting blood samples had the measurements of plasma ALA, LA, EPA, DHA, or AA. This may result in the potential sample selection bias in this study.

Third, we did not exclude any outliers in the analysis. Five participants were taking unusually high dosages of vitamin B6 supplements. The relative standard error (RSE) was 10.3% with the outliers included and 7.4% with the outliers excluded. The estimate with the outliers may still be considered reliable since the RSE did not exceed 20%. For these reasons, those unusually high total vitamin B6 intake values could be valid and therefore were retained in this study; however, these influential observations may introduce bias.

Fourth, the smaller sample size of the group of women (*n =* 380) than the group of men (*n =* 484) in the gender-stratified analysis might lead to larger sampling variation in women than in men, possibly influencing the results of the nonsignificant association between plasma PLP and plasma PUFA in women and the significant association in men.

Fifth a cross-sectional survey design only demonstrates statistical associations but does not provide causality, making it difficult to establish mechanisms underlying the observations in this study. Sixth, we only utilized plasma fatty acids since other sources of fatty acids, such as erythrocytes, were not available in the NHANES database; unavailability of other direct and functional vitamin B6 biomarkers in the 2003–2004 survey cycle makes analyses limited to plasma PLP.

Nevertheless, this study has strengths. First, the rich, multiethnic NHANES dataset allowed us to use multiple covariates, which may reduce potential sources of bias. Second, although the method of 24 h dietary recall to assess dietary information is subject to have measurement error associated with the dietary recall method (e.g., underreporting or overreporting of food intakes) [89], the 2 day non-consecutive 24 h recall used in this study is considered to perform better than a single 24 h recall since it allows for reducing intra-individual variability in nutrient intakes [90]. Third, since the 2003–2004 cycle is unique to provide data for vitamin B6 intake and plasma PLP and PUFA together, this study took an opportunity to evaluate the relationship between vitamin B6 intake and status and plasma PUFA in the US representative sample. 

In conclusion, the significant positive associations between plasma PLP and plasma EPA, DHA, EPA + DHA, EPA/AA, and (EPA + DHA)/AA were found in men only, not in women, among US young and middle-aged adults. Future large-scale prospective studies are necessary to confirm the relationship between gender, vitamin B6, and PUFA status. 

## Figures and Tables

**Table 1:** Demographic, socioeconomic, and other characteristics of participants by gender among US adults aged 20–59 y, NHANES 2003–2004.

	All (*n =* 864)		Men (*n =* 484)		Women (*n =* 380)		
Characteristics	*n*	% ± SE	*n*	% ± SE	*n*	% ± SE	*p* ^1^
Gender	864	100 ± 0	484	55.4 ± 1.5	380	44.6 ± 1.5	0.003
Age (years)							0.046
20–29	227	21.9 ± 1.5	147	25.3 ± 1.8	80	17.7 ± 2.1	
30–39	226	26.6 ± 1.6	125	26.1 ± 1.7	101	27.2 ± 3.1	
40–49	253	31.4 ± 1.7	128	27.8 ± 2.5	125	35.9 ± 2.4	
50–59	158	20.1 ± 2.1	84	20.8 ± 3.0	74	19.2 ± 2.0	
Race/Ethnicity							0.09
Non-Hispanic White	444	70.9 ± 3.7	245	70.6 ± 4.0	199	71.2 ± 3.7	
Non-Hispanic Black	180	10.4 ± 1.8	91	8.6 ± 1.8	89	12.6 ± 2.2	
Hispanic	200	13.0 ± 2.8	123	14.3 ± 3.3	77	11.4 ± 2.5	
Others	40	5.7 ± 0.5	25	6.4 ± 1.0	15	4.8 ± 1.0	
BMI (kg/m^2^)							0.0004
<18.5	12	1.6 ± 0.4	5	1.0 ± 0.4	7	2.5 ± 0.7	
18.5–24.9	264	31.5 ± 1.4	148	27.8 ± 2.2	116	36.2 ± 2.5	
25–29.9	294	34.9 ± 2.0	188	41.6 ± 2.0	106	26.5 ± 2.8	
≥30	283	32.0 ± 1.9	139	29.7 ± 2.3	144	34.9 ± 2.9	
PIR							0.0496
≤1.3	216	19.5 ± 1.7	108	16.6 ± 1.7	108	23.1 ± 2.8	
>1.3	612	80.5 ± 1.7	356	83.4 ± 1.7	256	76.9 ± 2.8	
Educational attainment							0.352
≤High school degree	430	43.1 ± 2.0	252	45.0 ± 2.0	178	40.7 ± 3.9	
>High school degree	434	56.9 ± 2.0	232	55.0 ± 2.0	202	59.3 ± 3.9	
Physical activity (MET min/week)							0.06
<500	343	35.7 ± 2.6	182	32.2 ± 3.2	161	40.0 ± 3.4	
500–1000	124	16.4 ± 1.6	66	15.3 ± 2.4	58	17.8 ± 1.8	
≥1000	397	47.9 ± 2.1	236	52.5 ± 2.9	161	42.3 ± 2.6	
Cigarette smoking							0.16
Never smoker	453	51.4 ± 2.4	235	48.0 ± 3.1	218	55.6 ± 3.8	
Former smoker	150	18.8 ± 2.0	92	21.1 ± 1.8	58	16.0 ± 3.3	
Current smoker	261	29.8 ± 2.1	157	30.9 ± 2.2	104	28.4 ± 3.0	
Alcohol consumption							0.002
Lifetime abstainer	89	9.1 ± 1.3	35	5.9 ± 1.0	54	13.2 ± 2.0	
Former drinker	112	14.1 ± 2.2	54	12.2 ± 2.7	58	16.6 ± 2.4	
Current drinker	588	76.8 ± 2.9	364	81.9 ± 3.1	224	70.2 ± 3.5	
Vitamin B6 supplement							0.018
No	619	66.8 ± 1.5	362	69.8 ± 1.8	257	63.1 ± 2.1	
Yes	243	33.2 ± 1.5	122	30.2 ± 1.8	121	36.9 ± 2.1	
n-3 PUFA supplement							0.36
No	846	97.1 ± 0.9	477	97.9 ± 1.0	369	96.2 ± 1.5	
Yes	16	2.7 ± 0.9	7	2.1 ± 1.0	9	3.8 ± 1.5	
Prescription medication							0.011
No	540	57.4 ± 2.0	332	62.3 ± 2.9	208	51.3 ± 2.5	
Yes	322	42.6 ± 2.0	152	37.7 ± 2.9	170	48.7 ± 2.5	
Menopausal status ^2^							<0.0001
No	NA	NA	NA	NA	297	78.1 ± 1.5	
Yes	NA	NA	NA	NA	83	21.9 ± 1.5	

BMI, body mass index; MET, metabolic equivalent of task; PIR, poverty income ratio; PUFA, polyunsaturated fatty acids; n, frequencies; %, sample-weighted percentages, SE, standard errors; NA, not applicable. Sample sizes were presented as unweighted. Values were expressed as sample-weighted percentages ± SE. ^1^ Rao-Scott F-adjusted chi-square tests for examining whether there are differences in proportions between men and women across categories of each characteristic. ^2^ Rao-Scott F-adjusted chi-square tests for examining whether there are differences in proportions between premenopausal and postmenopausal status. The supplement use of vitamin B6 and n-3 PUFA, respectively, was defined as a positive response to the question of taking any dietary supplements containing vitamin B6 and n-3 PUFA (eicosapentaenoic acid (EPA), docosahexaenoic acid (DHA), α-linolenic acid (ALA)) each in the past month.

**Table 2:** Distributions of the energy-adjusted intakes of vitamin B6 and PUFA and the plasma concentrations of PUFA and pyridoxal 5′-phosphate (PLP) by gender among US adults aged 20–59 years, NHANES 2003–2004.

	All (*n =* 864)			Men (*n =* 484)			Women (*n =* 380)			
	*n*	Mean ± SE		*n*	Mean ± SE		*n*	Mean ± SE		*p* ^1^
*Nutrient intakes from food*										
Total energy (kcal/d)	761	2375.2 ± 42.0		420	2763.2 ± 47.4		341	1896.1 ± 50.0		<0.0001
Vitamin B6 (mg/d)	761	2.05 ± 0.04		420	2.08 ± 0.06		341	2.01 ± 0.05		0.73
ALA (g/d)	761	1.64 ± 0.04		420	1.55 ± 0.03		341	1.75 ± 0.07		0.046
LA (g/d)	761	16.17 ± 0.40		420	15.68 ± 0.48		341	16.78 ± 0.40		0.38
EPA (g/d)	761	0.034 ± 0.003		420	0.039 ± 0.005		341	0.027 ± 0.003		0.022
DHA (g/d)	761	0.070 ± 0.005		420	0.078 ± 0.008		341	0.060 ± 0.004		0.027
AA (g/d)	761	0.15 ± 0.01		420	0.16 ± 0.01		341	0.14 ± 0.005		0.019
Total fat (g/d)	761	89.35 ± 1.27		420	87.08 ± 1.50		341	92.15 ± 1.37		0.06
*Nutrient intakes from food & supplements*										
Total vitamin B6 (mg/d)	761	5.44 ± 0.56		420	5.57 ± 0.69		341	5.28 ± 0.74		0.86
Total ALA (g/d)	761	1.65 ± 0.04		420	1.55 ± 0.03		341	1.76 ± 0.07		0.036
Total EPA (g/d)	761	0.037 ± 0.004		420	0.042 ± 0.006		341	0.031 ± 0.004		0.1
Total DHA (g/d)	761	0.072 ± 0.01		420	0.080 ± 0.008		341	0.063 ± 0.004		0.06
*Plasma Variables*										
ALA (μmol/L)	850	59.87 ± 1.27		475	60.26 ± 1.64		375	59.40 ± 1.62		0.99
LA (μmol/L)	850	3375.4 ± 35.0		475	3371.3 ± 40.6		375	3380.4 ± 36.4		0.9
EPA (μmol/L)	849	41.12 ± 1.10		474	43.13 ± 1.83		375	38.81 ± 0.91		0.09
DHA (μmol/L)	850	114.89 ± 3.77		475	112.71 ± 4.25		375	117.60 ± 3.58		0.043
AA (μmol/L)	850	758.70 ± 6.23		475	764.41 ± 11.74		375	751.82 ± 6.88		0.16
EPA+DHA (μmol/L)	849	158.73 ± 4.88		474	158.63 ± 6.16		375	158.86 ± 4.10		0.52
EPA/AA	849	0.054 ± 0.001		474	0.056 ± 0.002		375	0.052 ± 0.001		0.13
(EPA+DHA)/AA	849	0.209 ± 0.006		474	0.207 ± 0.006		375	0.211 ± 0.006		0.034
PLP (nmol/L)	854	42.35 ± 1.88		479	51.07 ± 2.61		375	33.52 ± 1.85		0.0002
PLP category ^2,3^										<0.0001
<20 nmol/L	175	18.6 ± 1.8		55	10.4 ± 1.9		120	29.0 ± 2.4		
≥20 nmol/L	679	81.4 ± 1.8		424	89.6 ± 1.9		255	71.0 ± 2.4		

AA, arachidonic acid; ALA, α-linolenic acid; DHA, docosahexaenoic acid; EPA, eicosapentaenoic acid; LA, linoleic acid; PLP, pyridoxal 5′-phosphate; PUFA, polyunsaturated fatty acids; *n*, frequencies; SE, standard error; %, sample-weighted percentages. Sample sizes were presented as unweighted. Values were expressed as arithmetic means or geometric means ± SE for continuous variables and sample-weighted percentages with SE for categorical variables. Log-transformed values of plasma PUFA and PLP were used for *t*-tests. Number of observations used for t-tests: *n =* 696 for nutrient intake variables; *n =* 683–684 for plasma PUFA; *n =* 675 for plasma PLP. ^1^ *t*-test for comparing the means of dependent variables between men and women. ^2^ Rao-Scott F-adjusted chi-square test for examining whether there are differences in proportions between men and women for the PLP category. ^3^ % ± SE. For nutrient intakes: adjusted for demographic variables (age, race/ethnicity), BMI, socioeconomic variables (PIR, educational attainment), physical activity level, cigarette smoking status, alcohol consumption, prescription medication use, menopausal status. For plasma PUFA: adjusted for demographic variables (age, race/ethnicity), BMI, dietary variables (total fat intake, total intakes of EPA, DHA, ALA, dietary intakes of LA and AA), socioeconomic variables (PIR, educational attainment), physical activity level, cigarette smoking status, alcohol consumption, prescription medication use, menopausal status. For plasma PLP: adjusted for demographic variables (age, race/ethnicity), BMI, dietary variables (total energy intake, total vitamin B6 intake), socioeconomic variables (PIR, educational attainment), physical activity level, cigarette smoking status, alcohol consumption, prescription medication use, menopausal status.

**Table 3:** Linear regression models: the association of plasma PLP concentration with plasma PUFA concentrations and ratios, stratified by gender among US adults aged 20–59 years, NHANES 2003–2004.

	Men (*n* = 484)				Women (*n* = 380)				
	β	b (95% CI)	R^2^	*p*	β	B (95% CI)	R^2^	*p*	*P*-*_interaction_*^1^
	**Plasma EPA** (µmol/L)								
Plasma PLP (nmol/L)									0.004
Model 0	0.203	0.155 (0.105, 0.204)	0.04	<0.0001	−0.0001	−0.0001 (−0.064, 0.064)	2.1 × 10^−8^	0.998	
Model 1	0.180	0.135 (0.079, 0.190)	0.24	0.0001	−0.048	−0.026 (−0.111, 0.059)	0.18	0.525	
Model 2	0.138	0.104 (0.055, 0.154)	0.27	0.0004	−0.052	−0.028 (−0.121, 0.065)	0.22	0.528	
	**Plasma DHA** (µmol/L)								
Plasma PLP (nmol/L)									0.020
Model 0	0.169	0.096 (0.032, 0.160)	0.03	0.006	−0.038	−0.016 (−0.090, 0.058)	0.001	0.65	
Model 1	0.165	0.094 (0.039, 0.148)	0.27	0.002	−0.055	−0.023 (−0.116, 0.069)	0.19	0.60	
Model 2	0.101	0.058 (0.004, 0.112)	0.31	0.036	−0.062	−0.026 (−0.122, 0.071)	0.23	0.58	
	**Plasma AA** (µmol/L)								
Plasma PLP (nmol/L)									0.365
Model 0	0.020	0.007 (−0.029, 0.043)	0.0004	0.70	−0.104	−0.028 (−0.057, 0.0005)	0.01	0.05	
Model 1	0.025	0.008 (−0.029, 0.046)	0.11	0.65	−0.095	−0.027 (−0.057, 0.004)	0.08	0.08	
Model 2	0.013	0.004 (−0.034, 0.042)	0.14	0.81	−0.085	−0.023 (−0.059, 0.012)	0.14	0.19	
	**Plasma EPA + DHA** (µmol/L)								
Plasma PLP (nmol/L)									0.010
Model 0	0.198	0.115 (0.060, 0.170)	0.04	0.001	−0.024	−0.010 (−0.077, 0.057)	0.001	0.76	
Model 1	0.186	0.108 (0.059, 0.156)	0.29	0.0003	−0.052	−0.021 (−0.109, 0.066)	0.19	0.61	
Model 2	0.125	0.073 (0.026, 0.121)	0.32	0.005	−0.058	−0.024 (−0.115, 0.068)	0.22	0.59	
	**Plasma EPA/AA ratio**								
Plasma PLP (nmol/L)									0.002
Model 0	0.215	0.147 (0.102, 0.191)	0.05	<0.0001	0.062	0.028 (−0.037, 0.094)	0.004	0.37	
Model 1	0.186	0.125 (0.079, 0.171)	0.29	<0.0001	0.001	0.001 (−0.074, 0.076)	0.16	0.99	
Model 2	0.144	0.099 (0.056, 0.142)	0.32	0.0002	−0.011	−0.005 (−0.092, 0.082)	0.20	0.90	
	**Plasma (EPA + DHA)/AA ratio**								
Plasma PLP (nmol/L)									0.004
Model 0	0.198	0.107 (0.055, 0.160)	0.04	0.001	0.054	0.018 (−0.043, 0.080)	0.003	0.53	
Model 1	0.181	0.098 (0.053, 0.144)	0.32	0.0003	0.016	0.005 (−0.064, 0.074)	0.19	0.87	
Model 2	0.123	0.068 (0.024, 0.113)	0.36	0.005	−0.001	−0.0004 (−0.070, 0.069)	0.22	0.99	

AA, arachidonic acid; DHA, docosahexaenoic acid; EPA, eicosapentaenoic acid; PLP, pyridoxal 5′-phosphate; PUFA, polyunsaturated fatty acids; b, unstandardized regression coefficient; β, standardized regression coefficients; 95% CI, 95% confidence intervals; *P-int*., *P*-interaction; R^2^, the coefficient of determination. Sample sizes are presented as unweighted. Plasma PUFA and PLP variables were log-transformed. Standardized coefficients (β) are to be interpreted as the change in log-transformed plasma PUFA concentrations and ratios in standard deviation (SD) for 1 SD of change in log-transformed plasma PLP concentration. For model 0, the unadjusted R^2^ is presented, and for models 1 and 2, the adjusted R^2^ is presented. Model 0: unadjusted. Model 1: adjusted for demographic variables (age, race/ethnicity), BMI, dietary variables (total fat intake, total intakes of EPA, DHA, and ALA, dietary intakes of LA and AA), menopausal status (for women). Model 2: adjusted for all variables in model 1 plus socioeconomic variables (PIR, educational attainment), physical activity level, cigarette smoking status, alcohol consumption, and prescription medication use. ^1^ *p*-value for the interaction term gender*PLP on plasma PUFA in the fully adjusted model 2. Number of observations used in the analysis of each model: Model 0: *n* = 469–470 for men, *n* = 370 for women; Model 1: *n* = 403–404 for men, *n* = 327 for women; Model 2: *n* = 380–381 for men, *n* = 294 for women.

## Data Availability

The data used for these secondary analyses of data are publicly available at the CDC webpage: https://wwwn.cdc.gov/nchs/nhanes/Default.aspx.

## Supplementary Materials

The following are available online at https://www.mdpi.com/2072-6643/13/2/477/s1, Table S1. Pearson correlation coefficients (ρ) between vitamin B6 intake and plasma PLP concentration by gender among US adults aged 20–59 years, NHANES 2003–2004, Table S2. Distributions of original-metric intakes of vitamin B6 and PUFA by gender among US adults aged 20–59 years, NHANES 2003–2004, Table S3. Distributions of serum iron and hemoglobin concentrations by gender among US adults aged 20–59 years, NHANES 2003–2004.
