# Supplementary material for: Gender Differences in the Associations of Plasma Pyridoxal 5′-Phosphate with Plasma Polyunsaturated Fatty Acids among US Young and Middle-Aged Adults: NHANES 2003–2004
Source: Nutrients. 2021 Jan 31;13(2):477. doi: 10.3390/nu13020477 (PMC7912414; doi:10.3390/nu13020477)
Supplement: Supplementary file 1 [file nutrients-13-00477-s001.pdf]

Supplementary Material

Table S1. Pearson correlation coefficients (ρ) between vitamin B6 intake and plasma PLP concentration by gender among US adults aged 20–59 y, NHANES 2003–2004

		**Total vitamin B6 intake** (mg/d)
		**ALL**
**Plasma PLP** (nmol/L)	**ρ (rho)**	0.26
	P [1]	<.0001
	n	751
		**Men**
**Plasma PLP** (nmol/L)	**ρ (rho)**	0.24
	P [1]	<.0001
	n	415
		**Women**
**Plasma PLP** (nmol/L)	**ρ (rho)**	0.30
	P [1]	<.0001
	n	336

- PLP, pyridoxal 5′-phosphate; *n*, frequencies.
- Log-transformed values of plasma PLP were used for Pearson correlation tests.
- [1] P-values for Pearson correlation test.
- Total number of observations: *n*=864 (484 men; 380 women)

Table S2. Distributions of original metric intakes of vitamin B6 and PUFA by gender among US adults aged 20–59 y, NHANES 2003–2004

**Original metric**	**All** (*n*=864)		**Men** (*n*=484)		**Women** (*n*=380)		
	n	**Mean ± SE**	***n***	**Mean ± SE**	***n***	**Mean ± SE**	***P*** [1]
Nutrient intakes from food							
Vitamin B6 (mg/d)	761	2.05 ± 0.04	420	2.34 ± 0.07	341	1.69 ± 0.07	0.59
ALA (g/d)	761	1.64 ± 0.05	420	1.79 ± 0.05	341	1.45 ± 0.08	0.017
LA (g/d)	761	16.17 ± 0.43	420	18.10 ± 0.52	341	13.80 ± 0.52	0.36
EPA (g/d)	761	0.03 ± 0.003	420	0.04 ± 0.01	341	0.02 ± 0.003	0.08
DHA (g/d)	761	0.07 ± 0.01	420	0.08 ± 0.01	341	0.05 ± 0.004	0.11
AA (g/d)	761	0.15 ± 0.01	420	0.18 ± 0.01	341	0.12 ± 0.005	0.011
Total fat (g/d)	761	89.35 ± 2.36	420	102.51 ± 2.59	341	73.09 ± 2.80	0.031
Nutrient intakes from food & supplements							
Total vitamin B6 (mg/d)	761	5.44 ± 0.56	420	5.61 ± 0.69	341	5.23 ± 0.75	0.79
Total ALA (g/d)	761	1.65 ± 0.05	420	1.80 ± 0.05	341	1.46 ± 0.08	0.003
Total EPA (g/d)	761	0.04 ± 0.004	420	0.05 ± 0.01	341	0.03 ± 0.004	0.009
Total DHA (g/d)	761	0.07 ± 0.01	420	0.09 ± 0.01	341	0.05 ± 0.004	0.003

- AA, arachidonic acid; ALA, α-linolenic acid; DHA, docosahexaenoic acid; EPA, eicosapentaenoic acid; LA, linoleic acid; PUFA, polyunsaturated fatty acids; *n*, frequencies; SE, standard error; %, sample-weighted percentages.
- Sample sizes were presented as unweighted. Values were expressed as means ± SE for continuous variables.
- Number of observations used for *t*-tests: *n*=696 for nutrient intake variables
- [1] *t*-tests for comparing the means of dependent variables between men and women.
- Adjusted for demographic variables (age, race/ethnicity), BMI, socioeconomic variables (PIR, educational attainment), physical activity level, cigarette smoking status, alcohol consumption, prescription medication use), menopausal status, total energy intake

Table S3. Distributions of iron intake, serum iron and hemoglobin concentrations by gender among US adults aged 20–59 y, NHANES 2003–2004

	**All** (*n*=864)		**Men** (*n*=484)		**Women** (*n*=380)		
	n	**Mean ± SE**	***n***	**Mean ± SE**	***n***	**Mean ± SE**	***P*** [1]
Nutrient intake variables							
Original metric							
Nutrient intakes from food							
Iron (mg/d)	761	16.94 ± 0.46	420	19.57 ± 0.69	341	13.70 ± 0.48	0.84
Nutrient intakes from food & supplements							
Total Iron (mg/d)	761	19.87 ± 0.49	420	22.02 ± 0.53	341	17.21 ± 0.82	0.001
Energy-adjusted							
Nutrient intakes from food							
Iron (mg/d)	761	16.94 ± 0.47	420	17.05 ± 0.68	341	16.82 ± 0.41	0.77
Nutrient intakes from food & supplements							
Total Iron (mg/d)	761	19.87 ± 0.55	420	19.53 ± 0.61	341	20.28 ± 0.75	0.22
Plasma variables							
Iron, serum (μmol/L)	859	14.78 ± 0.26	483	16.89 ± 0.33	376	12.51 ± 0.24	<.0001
Serum iron category [1,3]							<.0001
Low	189	20.11 ± 1.46	74	14.17 ± 1.57	115	27.52 ± 1.97	
High	670	79.89 ± 1.46	409	85.83 ± 1.57	261	72.48 ± 1.97	
Hemoglobin (g/dL)	864	14.69 ± 0.08	484	15.66 ± 0.07	380	13.57 ± 0.08	<.0001
Iron deficiency anemia [2,3]							0.0003
Yes	45	4.13 ± 0.82	3	0.73 ± 0.49	42	8.33 ± 1.64	
No	819	95.88 ± 0.82	481	99.27 ± 0.49	338	91.67 ± 1.64	

- *n*, frequencies; SE, standard error; %, sample-weighted percentages.
- Sample sizes were presented as unweighted. Values were expressed as geometric means ± SE for continuous variables and sample-weighted percentages ± SE for categorical variables.
- Log-transformed values of serum iron and hemoglobin were used for *t*-tests.
- [1] *t*-test for comparing the means of dependent variables between men and women.
- [2] Rao-Scott F-adjusted chi-square test for examining whether there are differences in proportions between men and women across categories of each characteristic.
- [3] % ± SE
- For nutrient intake: Adjusted for demographic variables (age, race/ethnicity), BMI, socioeconomic variables (PIR, educational attainment), physical activity level, cigarette smoking status, alcohol consumption, prescription medication use, menopausal status, total energy intake (only for original metric dietary variables)
- Plasma variables: Adjusted for demographic variables (age, race/ethnicity), energy-adjusted total iron intake, BMI, socioeconomic variables (PIR, educational attainment), physical activity level, cigarette smoking status, alcohol consumption, prescription medication use, menopausal status.
- Low iron serum level is defined as <12 μmol/L for men and <10 μmol/L for women [9].
- Iron deficiency anemia is defined as hemoglobin <13 g/dL for men and hemoglobin <12 g/dL for women.
